# Bioengineering of Humanized Bone Marrow Microenvironments in Mouse and Their Visualization by Live Imaging

**DOI:** 10.3791/55914

**Published:** 2017-08-01

**Authors:** Diana Passaro, Ander Abarrategi, Katie Foster, Linda Ariza-McNaughton, Dominique Bonnet

**Affiliations:** ^1^Haematopoietic Stem Cell Laboratory, The Francis Crick Institute

**Keywords:** Developmental Biology, Issue 126, Live imaging, two-photon microscopy, bone marrow, stroma, microenvironment, humanized niche, human hematopoietic stem cells, human leukemia, vasculature, endothelial cells, mesenchymal stromal cells, BMP2

## Abstract

Human hematopoietic stem cells (HSCs) reside in the bone marrow (BM) niche, an intricate, multifactorial network of components producing cytokines, growth factors, and extracellular matrix. The ability of HSCs to remain quiescent, self-renew or differentiate, and acquire mutations and become malignant depends upon the complex interactions they establish with different stromal components. To observe the crosstalk between human HSCs and the human BM niche in physiological and pathological conditions, we designed a protocol to ectopically model and image a humanized BM niche in immunodeficient mice. We show that the use of different cellular components allows for the formation of humanized structures and the opportunity to sustain long-term human hematopoietic engraftment. Using two-photon microscopy, we can live-image these structures *in situ* at the single-cell resolution, providing a powerful new tool for the functional characterization of the human BM microenvironment and its role in regulating normal and malignant hematopoiesis.

**Figure:**
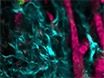


## Introduction

Cell fate decisions observed in stem cell compartments are tightly regulated by both intrinsic and extrinsic factors. In particular, it is now widely recognized that the BM microenvironment plays a fundamental role in controlling the switch in HSCs from a quiescent to an active state, as well as in their self-renewal or differentiation fate decision^1^. Moreover, recent findings indicate that hematological malignancies affect the function of the BM microenvironment, pointing to the existence of active crosstalk between the two compartments^2^,^3^,^4^,^5^,^6^. Despite recent advances, many key questions remain about how the activity of specific BM-niche components contribute to HSC behavior and malignant transformation.

The BM microenvironment is a highly heterogeneous and complex mixture of many different cell types, each with specialized functions. The abundant endothelial (EC) and vascular component regulate nutrient and metabolite turnover, the ingress and egress of different cells to and from the BM, and several HSC functions^7^,^8^. Mesenchymal stromal cells (MSCs), a heterogeneous population of undifferentiated stem cells and progenitors committed through three different lineages (*i.e., *osteogenic, chondrogenic, an adipogenic), are another fundamental component of the BM niche. These MSCs localize both in central areas of the BM and in the proximity to the endosteal region. They may be associated with vascular structures and are implicated in the regulation of HSC function^9^,^10^,^11^,^12^,^13^,^14^,^15^.

Many reports suggest that HSCs reside in various defined sites within the marrow and that their function might depend upon their precise localization. Most of the present knowledge regarding HSCs and their interaction with the BM microenvironment derives from murine studies^1^. The use of xenograft models has extended this knowledge to human normal and malignant HSCs, engrafting within the murine BM of immunodeficient mice^16^,^17^,^18^,^19^,^20^. Although this represents a valid model, it still presents many challenges, such as the need to condition the recipient mouse in most cases to allow for human HSC homing and engraftment or the cross-species barrier and its poorly understood influence on cell-cell interactions and functions.

The use of neutralizing antibodies and genetically modified mice, along with xenotransplantation, has been instrumental in highlighting the complex dialog that human HSCs establish with their microenvironments. The introduction and development of intravital two-photon confocal microscopy has moved these studies a step forward, allowing for the direct, high-resolution, and dynamic imaging of the bone marrow^19^,^20^,^21^,^22^ and provides a powerful tool for the functional characterization of the BM microenvironment and its role in regulating HSC function. In order to circumvent some of the problems arising in classical xenotransplantation models, the concept of engineering a humanized BM structure has been brought to the fore. Merging biomaterials and cell-implantation concepts, reports have shown the feasibility of mimicking the human bone marrow microenvironment in heterotopic regions^23^,^24^,^25^,^26^,^27^. This opens the possibility of using bioengineering in mouse models to study human normal and malignant hematopoiesis^28^,^29^,^30^,^31^,^32^,^33^,^34^,^35^,^36^,^37^,^38^,^39^, tumorigenesis, and metastasis^40^,^41^,^42^,^43^,^44^.

Based on previous experience in bone tissue engineering and *in vivo* imaging^19^,^22^,^45^,^46^,^47^,^48^,^49^,^50^,^51^,^52^, we describe a protocol to bioengineer and live-image organotypic human BM tissues. These structures originate from the implantation of human BM-derived stromal cells into collagen-based scaffolds subcutaneously grafted in immunodeficient mice. In a previous report, we demonstrated that human MSCs ensure the formation of a human microenvironment adequate for the engraftment of human hematopoietic cells^45^. Furthermore, here we describe how the co-implantation of other human BM cellular components, such as human endothelial cells (hEC), and/or cytokines important for bone formation (*e.g., *hBMP2), cooperate with hMSCs to generate different humanized microenvironments, which can be live-imaged *in situ*.

## Protocol

All animal experiments were performed under PPL 70/8904, approved by the U.K. Home Office and in accordance with Cancer Research UK guidelines. The use of human umbilical cord blood (UCB) and primary human acute myeloid leukemia (AML) samples was approved by the East London Ethical Committee after receiving consent and was carried out in accordance with the Declaration of Helsinki.

### 1. Bioengineering Collagen-based Scaffolds with Human Hematopoietic and Stromal Cells

NOTE: The entire protocol should be performed in sterile conditions and with sterile material. Cell culture medium 1 corresponds to hMSC medium (MEM-α, P/S, and 10% hMSC-FBS); cell culture medium 2 corresponds to EC medium (M199, 20% FBS, P/S, 10mM HEPES, 50 µg/mL heparin, 2 mM glutamine and 50 μg/mL ECGS) and cell culture medium 3 corresponds to hematopoietic cell medium (H5100 and P/S).

1. Prepare umbilical cord blood mononuclear cells (CB-MNCs) or primary AML (bone marrow or peripheral blood MNCs) using a Ficoll-Paque density gradient, according to well-established protocols^53^.
  1. For AML, deplete the T cells using OKT.3 antibody. Incubate 4 µg of OKT.3 per 1 x 10^6^ AML MNCs for 30 min at room temperature before washing the cells in PBS. If required, cryopreserve the MNCs in FBS with 10% DMSO at 2 x 10^8^ cells per mL.
2. Prepare hMSC (culture medium 1) and EC (culture medium 2) cell cultures. Plate hMSC cells (see the **Table of Materials**) on regular cell culture flasks in hMSC medium. Plate ECs (see the **Table of Materials**) on collagen 1-coated surfaces in EC medium.
3. At 85-90% cell confluence remove the medium, wash twice with PBS, and add trypsin-EDTA solution (20 µL per cm^2^).
  1. After 5 min, check that cells are detached. Recover the cells by diluting 1:3 with cell culture medium. Centrifuge at 300 x g for 5 min and re-suspend in the corresponding medium for each cell type and count the cells using a Neubauer chamber.
  2. Use cells at 2 x 10^6 ^- 10^7^ cells per mL. If both cell types need to be used together, mix the hMSC and EC suspensions in a 1:1 ratio.
4. Transfer the cell suspension to an insulin syringe.
5. Using a scalpel, cut the sterilized gelatin sponge (*e.g.,* gelfoam) initial scaffold (20 mm x 60 mm x 7 mm) into 24 pieces of similar size (6.6 mm x 7.5 mm x 7 mm; Figure 1A and **B**).
6. Reconstitute gelatin scaffolds by immersion in PBS (5 min).
7. One by one, gently put the scaffolds on a sterile tissue to remove excess PBS. Transfer the scaffolds to one well of a 24-well plate (ultra-low attachment surface) and use the syringe to inject 50 µL containing 10^5^ - 10^6^ cells (hMSC alone or in combination with hEC; Figure 1C).
8. Repeat step 1.7 with each scaffold until all required scaffolds are seeded with stromal cells.
9. Incubate for 1 h inside a cell culture incubator (37 °C and CO_2_ 5%).
10. Fill each well with 3 mL of cell culture medium 1 (Figure 1D) and return the scaffolds to a cell culture incubator (37 °C and CO_2_ 5%) for overnight incubation. Use cell culture medium 2 if ECs are used in the scaffolds.
11. Thaw CB-MNCs and isolate CD34^+^ cells from them following an appropriate CD34^+^ section kit protocol. Suspend the selected CD34^+^ cells in medium 3 and count them in a Neubauer chamber. NOTE: Here, cells were used at a concentration of 2 x 10^6^ cells per mL.
  1. If AML patient-derived primary samples are used, thaw the cells, dilute them 1:10 in FBS, centrifuge them for 5 min at 300 x g, resuspend the cells in PBS-2% FBS, add anti-human CD3 antibody (2 - 4 µg per 10^6^ cells), and incubate at room temperature for 30 min. Centrifuge for 5 min at 300 x g and resuspend in hematopoietic cell medium supplemented with cytokines (20 ng/mL granulocyte-colony stimulating factor (G-CSF), 20 ng/mL IL-3, and 20 ng/mL thrombopoietin (TPO)).
12. Repeat steps 1.7 - 1.9 but in this case seed 1 x 10^5^ human hematopoietic cells instead of stromal components, as above.
  1. Fill the well with 3 mL of cell culture medium 3 (Figure 1D) and return the scaffolds to a cell culture incubator (37 °C and CO_2_ 5%) for overnight incubation. Use a 1:1 mix of cell culture media 2 and 3 If ECs are used in the scaffolds.
13. For only recombinant human bone morphogenetic protein-2 (rhBMP-2) carrier scaffolds, gently put the scaffolds one by one on a sterile tissue to remove excess PBS. Transfer the scaffolds to one well of a u-bottom, 96-well plate (Figure 1E) and add 5 µL of rhBMP-2, thoroughly covering the scaffold.
  1. Add 20 µL of thrombin followed by 20 µL of fibrinogen, thoroughly covering the scaffold each time (Figure 1F). Repeat the procedure for each scaffold until all required scaffolds are treated and then incubate for 5 - 10 min at 37 °C. Check if coagulation has successfully formed.

### 2. Surgical Implantation of Bioengineered Scaffolds

NOTE: Here, either male or female, 6- to 12-week-old NSG mice were used. As the animals are immunodeficient, all procedures should be done in sterile conditions. Steps 2.10 - 2.13 are related to survival strategies and post-surgical care.

1. 60 - 120 min before surgery, subcutaneously administer pain medication (carprofen, 5 mg/kg bodyweight/mouse).
2. Induce anesthesia in a chamber with 0.5% isoflurane and 2 L/min O_2_.  Mice should be monitored continuously during the procedure. Transfer the animal to the surgical area in prone position in order to have easy access to the back. Keep the animal under anesthesia using a nose cone supplying 1.5% isoflurane and 2 L/min O_2_. Keep the mouse under anesthesia during the surgical procedure and frequently check the animal state.
3. While it is under anesthesia, use ophthalmic gel on the eyes of the mouse to prevent dryness, and keep the mouse at 37 °C.
4. Shave the surgical area on the back of the mouse using an electric trimmer. To sterilize the skin, dip a cotton tip in diluted clorexidine (diluted 1:10 in PBS) and use this tip to clean the skin surface. Repeat this procedure twice.
5. Using sterile forceps and a scalpel (or scissors), make a 0.5- to 0.7-cm anterior-to-posterior full incision of the skin. Use the forceps inserted under the subcutaneous tissue to make a pocket.
6. Insert the scaffold subcutaneously, making sure that it is placed deep within the pocket (Figure 1G and **H**). Close the incision with surgical glue (Figure 1I and **J**).
7. To treat post-surgical pain, subcutaneously administer buprenorphine (0.1 mg/kg bodyweight).
8. During recovery, place the animal on its side in a pre-warmed cage and monitor recovery until normal behavior is observed.
9. Dilute pain medication in water (carprofen, 0.1 mg/mL of water) and provide it to animals as drinking water for 4 days after surgery.
10. Check the animal and the wound frequently during the 48-h post-surgery period for possible adverse effects.

### 3. Mouse Treatments, Euthanasia, and Sample Retrieval for Imaging

NOTE: Analysis of the scaffolds is performed between 8 and 24 weeks post-implantation.

1. 60 min before imaging, keep the implanted mouse warm in a heating box at 37 °C and intravenously administer 100 µL of human immunoglobulin to block unspecific sites.
2. 30 min before imaging, intravenously administer 10 µg (per mouse) of specific antibodies to label the cells of interest.
3. 5 min before imaging, intravenously administer 15 µL (diluted in 100 µL of PBS) of 655-nm fluorophore-labeled, vessel-pooling agent (655-VPA) to visualize vascular structures.
4. Euthanize the mouse via cervical dislocation.
5. Using sharp scissors, make a longitudinal skin incision on the back of the mouse, near the original implantation site.
6. With the help of tweezers and scissors, carefully separate the skin from the subcutaneous pocket where the scaffold has been implanted.
7. Hold the scaffold with tweezers and gently explant it from the skin by cutting the residual membrane and tissue surrounding the scaffold using scissors. See examples of scaffolds to be recovered in Figure 2.
8. Secure the scaffold with fast-acting adhesive glue to an imaging plate (a 35 mm x 10 mm Petri dish) and fill with saline solution (PBS) at room temperature.
9. For BMP scaffolds, before filling the plate with PBS, use a surgical microdrill to thin the bone surface under a microsurgical microscope; this allows fluorophore visualization and high-resolution image capture. Use either the 1.2- or 1.6-mm burrs, depending upon the size of the scaffold. NOTE: The user will realize how much to drill depending upon the thickness of the bone. In general, as the BMP scaffolds are vascularized, the bone will slightly change color and become more red when approaching the correct thickness for imaging.
10. Insert the plate onto the stage of the confocal microscope.

### 4. Live-imaging Using Two-photon Microscopy

NOTE: When using non-descanned detectors (NDD), always use the NDD slider for imaging to direct the fluorescence to the NDD. The microscope configuration is provided in Figure 3.

1. Switch on the microscope and the computer, start the software by clicking "Start System," and go to the "Acquisition" mode.
2. Tick the "Show manual tools" box. In the "Laser" menu switch "on" the two-photon laser and allow it to warm up and stabilize.
3. In the "Imaging Setup" menu, simultaneously activate "Channel Mode" and "Switch track every Frame." In the "Light Path" menu, select "Non Descanned'" and "Main Beam Splitter MBS 760+." Tick to activate the four NDDs and set the configuration as illustrated in Figure 3. NOTE: With this configuration, the collagen signal from bone structures (second harmonic generation, SHG) is collected at 380 - 485 nm, FITC-hCD31+ human endothelial cells and AF488- hCD45+ human hematopoietic cells at 500-550 nm, and 655-VPA at 640 - 690 nm.
4. In the "Channels" menu, set the "laser wavelength" to 890 nm and the power to 50%. Set the "Gain (Master)" to 500-600, the "Digital Offset" to 0, and the "Digital Gain" to 15 for each channel. Adjust these values once the acquisition has started.
5. In "Acquisition Mode," set up the required parameters to obtain high-resolution images without damaging the tissue and bleaching the fluorophores. Set "Scan Mode" to "Frame," "Frame Size" to "x512 y512," Line Step" to 1, "Speed" to 9, "Averaging number" to 8, "Bit Depth" to "8 Bit," "Mode" to "line," "Direction" to "bidirectional," and "Method" to "mean."
  1. Set the "Zoom" to 1 for the initial scan of the image, and increase it if required to focus on particular areas.
6. Place the plate containing the scaffold on the microscope stage under the 20X, 1.0 NA water-immersion lens and lower the lens until it touches the saline solution. Set the focus of the lens on the scaffold using the microscope eyepieces, using a lamp as the light source.
7. Activate the "Z-stack" menu, select the "First/Last" function, and set the required interval between two sub-sequential slices (*e.g.,* 2-µm Z-stack). Keep the intervals constant within the Z-stack.
8. Select "Live" to image a live scan of the sample and adjust the "Digital Gain" and "Digital Offset" for optimal exposure. To visualize multiple channels at the same time, select the "Split" function.
9. In the "Stage" menu, while in "Live" mode, scan the image and "Mark" regions of interest (ROI), such as the location of human hematopoietic cells and vascular structures. When the scan of the sample is complete, move to the first ROI to start imaging.
10. In "Live" mode, set the top and bottom of the 3D Z-stack surrounding the area of interest using the "Set first" and "Set last" functions. Once finished, set the center, "C." Start the acquisition of the ROI with the "start experiment" button. See examples of images in Figure 4, Figure 5, and Figure 6.
11. Once acquisition is complete, save the image in the designated folder. Move to the next ROI and repeat step 4.10. Once the experiment is complete, remove the imaging plate from the microscope, carefully detach the sample from the plate, and clean any residual glue.
12. Prepare the sample for the next analysis technique.

### 5. Sample Processing for Histology and Immunostaining

NOTE: Samples are processed according to the protocol described in the JoVE general laboratory techniques^54^ describing sample fixation, embedding, and sectioning processes. Bone-forming samples should be treated for 7 days in an EDTA-based decalcifying agent between the fixation and embedding processes. The blocking/permeabilization solution is 10 mM PBS pH 7.4 buffer with 1% Triton X-100, 1% bovine serum albumin (BSA), and 10% normal goat serum (NGS).

1. Put the slices in xylene for 10 min), xylene for 5 min, 100% ethanol for 5 min, 70% ethanol for 5 min, 50% ethanol for 5 min, and H_2_O for 5 min.
2. Transfer the slices to a citrate-based antigen unmasking working solution.
3. Boil the slices for 15 min and allow them to cool to room temperature.
4. Wash the slices in 10 mM PBS pH 7.4 solution with 1% Triton X-100 (5 min, 3 times).
5. Transfer the samples to the blocking/permeabilization solution and incubate for 30 min.
6. Add primary antibody diluted in blocking/permeabilization solution and incubate overnight at 4 °C.
7. Wash the slices in 10 mM PBS pH 7.4 solution with 1% Triton X-100 (5 min, 3 times).
8. Add secondary antibody diluted in blocking/permeabilization solution for 1 h in the dark at room temperature.
9. Wash with H_2_O (5 min, 3 times).
10. To reduce background fluorescence, immerse the slices in Sudan Black working solution for 10 min, in the dark and at room temperature.
11. Wash the slices in H_2_O (5 min, 3 times).
12. Mount the slices using fluorescent mounting medium with DAPI (0.5 µg/mL).
13. Store the slices at 4 °C and check that the mounting medium is dry before conducting fluorescent imaging. See example images in Figure 7.

## Representative Results

In Figure 1, representative images of the scaffold cell seeding and implantation processes are shown. In Figure 1C, note that cells are injected directly into the scaffold. In Figure 1G, note that an incision is made in the back of the mouse, where the subcutaneous pocket is created and the scaffold is implanted. Figure 2 shows the gross morphology of different scaffolds implanted in NSG mice and retrieved after 8 weeks. Note the slight vascularization in hMSC seeded scaffolds (Figure 2A). The co-seeding of human ECs with hMSCs in the scaffold allows for the formation of more relevant vasculature in scaffolds (Figure 2B). Finally, the presence of rhBMP-2 induces bone formation. The retrieved scaffolds are bigger in this case, and they are constituted by bone-resembling hard tissue.

Figure 3 shows the channel configuration setup on the microscope for live-imaging with NDD (details in the figure legend). Figure 4 and **Video 1** show human hematopoietic cells in hMSC-coated scaffolds. Scaffolds were explanted 8 weeks post-implantation and after the intravenous inoculation of AF488-hCD45 antibody and 655-VPA. This procedure allows for the visualization of implanted human hematopoietic cells and the vascular structure by two-photon confocal microscopy. In this case, the images show blood vessels (655-VPA) in scaffolds and the long-term engraftment of human hematopoietic cells (AF488-hCD45) in the scaffold parenchyma. Figure 5 and **Video 2** correspond to human scaffolds seeded with hECs and hMSCs. 8 weeks after surgery, scaffolds were explanted after the intravenous inoculation of FITC-hCD31 antibody and 655-VPA, and images were acquired with a two-photon confocal microscope, as mentioned before. Images show the participation of hECs in vessel formation in the scaffold, resulting in a murine-human chimeric vasculature.

Figure 6A shows representative data of the approach used to stimulate bone formation in MSC scaffolds. Similar to previous figures, 8 weeks after implantation, the intravenous inoculation of 655-VPA was performed, scaffolds were retrieved, and images were acquired with two-photon confocal microscopy. rhBMP-2-stimulated scaffolds induce the formation of bone tissue, which could be visualized due to the SHG (cyan color in the images) provided by the calcium in the bone. The provided images also show the formation of cavities and vascularized endosteal tissue, which highly resemble the BM endosteal tissue. In Figure 6B and **Video 3**, hECs were co-implanted with hMSCs. Scaffolds were retrieved after the intravenous inoculation of FITC-hCD31 antibody and 655-VPA, and two-photon confocal microscopy images show the participation of hECs in the neovascularization in a bone-forming scaffold.

Figure 7 shows representative images of histology, a procedure performed to corroborate previously described results. Immunofluorescent images show mouse vasculature, hECs, hMSCs, and long-term engrafted human hematopoietic cells in the scaffold structures. Scaffolds retrieved from mice were fixed and used for immunofluorescence. In the rhBMP-2 carrier bone-forming scaffolds (Figure 7D**-F**), note the morphology of the tissue, resembling mature bone marrow with adipose tissue. In this bone-forming scaffold, we show that hMSCs are fibroblasts, which would indicate that they contribute to newly formed tissue as stromal cells. We also show human adipocyte marker expression, which would indicate that hMSCs also contribute to adipose tissue formation.

**Figure:**
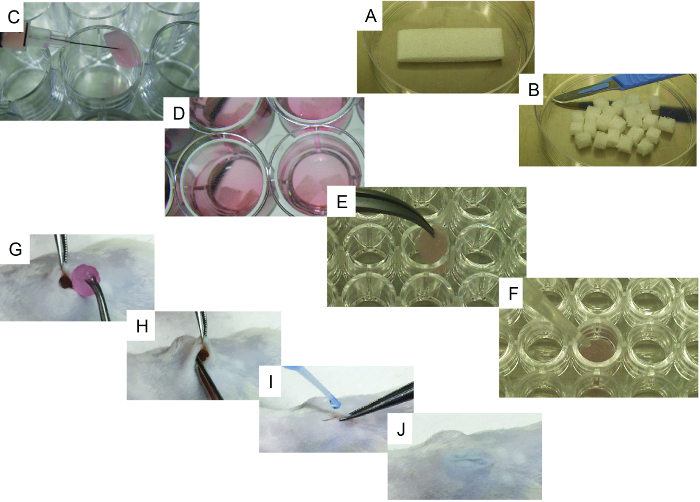

**Figure 1. Representative Images of the Cell-seeding and Implantation Processes. A) **Initial scaffold and its cutting method using a scalpel. **B)** 24 pieces obtained from the initial scaffold. **C) **Scaffold cell-seeding method using a syringe. **D)** Cell-seeded scaffolds with culture medium, ready to be implanted. **E-F) **Specific steps for bone-forming scaffolds: **E) **scaffold being transferred to a 96-well, u-bottom plate and **F)** representative image of the method used to add rhBMP2, thrombin, and fibrinogen to the scaffold. **G-J)** Surgical implantation procedure under general anesthesia: **G) **wound created in the skin and scaffold implantation, **H)** implantation method, and **I-J) **wound closing procedure using surgical glue. Please click here to view a larger version of this figure.

**Figure:**
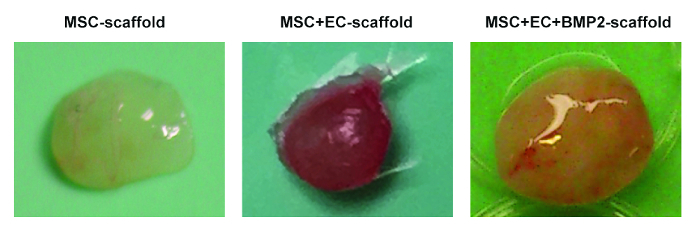

**Figure 2. Different Scaffolds Retrieved from Mice.** Representative images of MSC scaffolds (left), MSC+EC scaffolds (middle), and MSC+EC+BMP scaffolds (right). Please click here to view a larger version of this figure.

**Figure:**
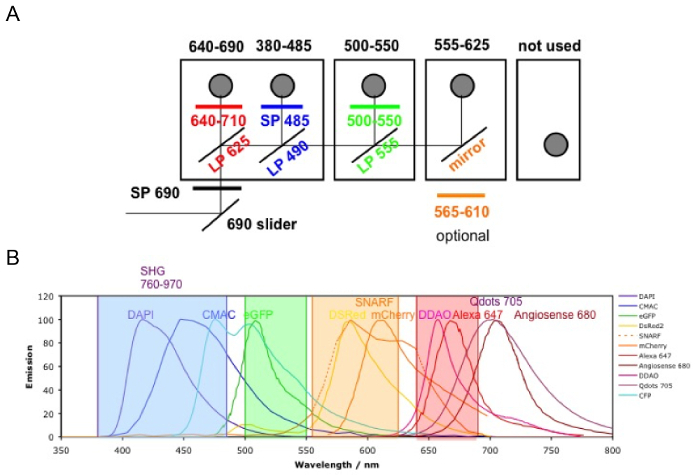

**Figure 3. Channel Configuration.** The microscope filter setup is shown. **A) **There are four NDD detector modules: in the first module, there are two filter cubes; the second and third modules have one filter cube each; and the last module has no cube (not used). The first photomultiplier tube (PMT) is for the far-red channel (640 - 690 nm), reflecting the lower wavelengths; the following ones are 380 - 485 nm, 500 - 550 nm, and 555 - 625 nm (the order is always from the lower to higher wavelengths). **B) **The fluorophore emissions detectable with the above configurations (color coded). Please click here to view a larger version of this figure.

**Figure:**
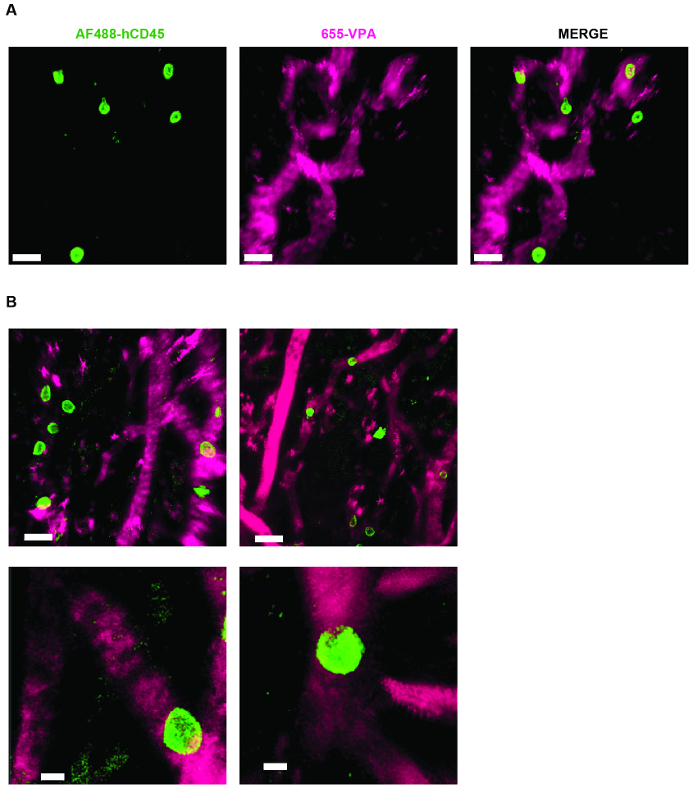

**Figure 4. MSC Scaffolds Allow for the Formation of a Niche for Human Hematopoietic Cells.A) **and** B)** 3D reconstructions of Z-stacks taken post-explant, after intravenous inoculation with AF488-hCD45 (green), to label human hematopoietic cells, and 655-VPA (magenta)m to label vasculature. Scale bars represent 20 µm in A and B (upper panels) and 5 µm in B (lower panels). Please click here to view a larger version of this figure.


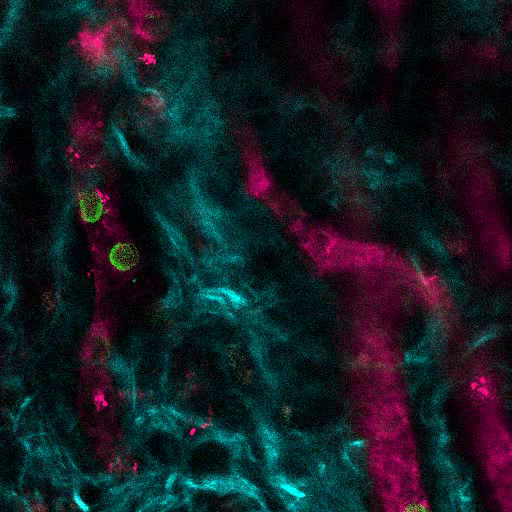
**Video 1. MSC Scaffolds Allow for the Formation of a Niche for Human Hematopoietic Cells**. 3D reconstruction of human hematopoietic cells (AF488-hCD45) associated with the vasculature (655-VPA) in the MSC scaffold (collagen structures: SHG, cyan). Each stack measures 140 x 140 µm. Please click here to view this video. (Right-click to download.)

**Figure:**
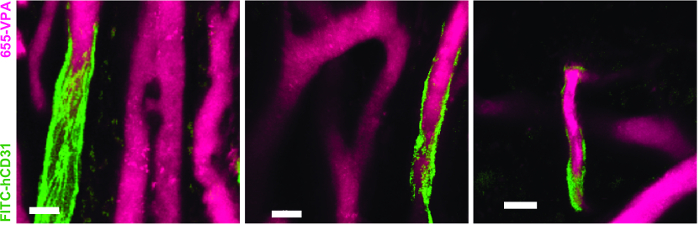

**Figure 5. Human ECs Participate in the Formation of Humanized Vessels in MSC Scaffolds.** 3D reconstruction of vasculature (655-VPA) lined by ECs of human origin (FITC-hCD31) in MSC+EC scaffolds. Scale bars represent 20 µm. Please click here to view a larger version of this figure.


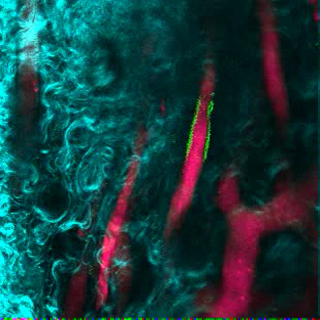
**Video 2. Human ECs Participate in the Formation of Humanized Vessels in MSC Scaffolds.** 3D reconstruction of human ECs (FITC-hCD31) lining the vasculature (655-VPA) in MSC+EC scaffolds. Each stack measures 240 x 240 µm. Please click here to view this video. (Right-click to download.)

**Figure:**
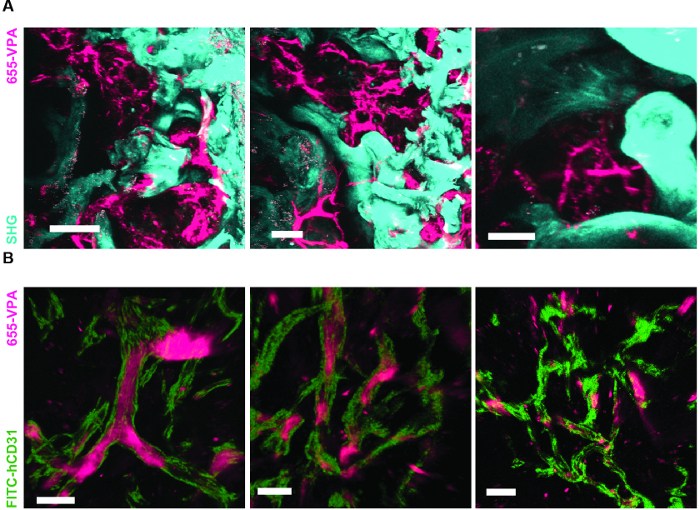

**Figure 6. rhBMP-2 Carrier Scaffolds have Bone Surfaces and Humanized Vasculature Similar to the Bone Marrow.A)** 3D reconstruction of MSC+BMP scaffolds showing the formation of bone structures (SHG-cyan) and vasculature (655-VPA). Scale bars represent 100 µm (left), 70 µm (middle), and 50 µm (right). **B)** 3D reconstruction of MSC+EC+BMP scaffolds showing humanized vessels (655-VPA) lined with human ECs (FITC-hCD31). Scale bars represent 50 µm (left) and 30 µm (middle and right). Please click here to view a larger version of this figure.


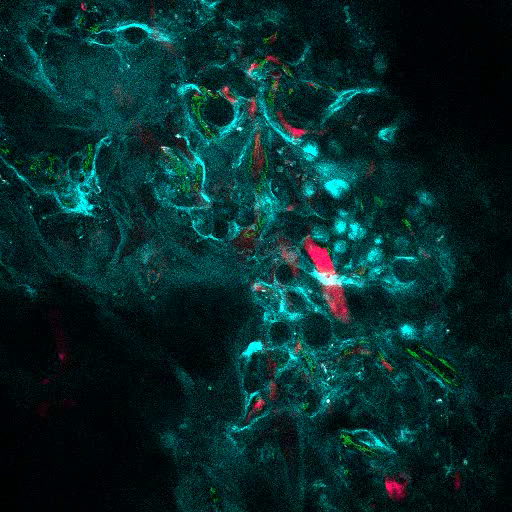
**Video 3. rhBMP-2 Carrier Scaffolds have Bone Surfaces and Humanized Vasculature Similar to the Bone Marrow. **3D reconstruction of MSC+VERA+BMP scaffold (bone: SHG; vessels: 655-VPA; human ECs: FITC-hCD31). Each stack measures 600 x 600 µm. Please click here to view this video. (Right-click to download.)

**Figure:**
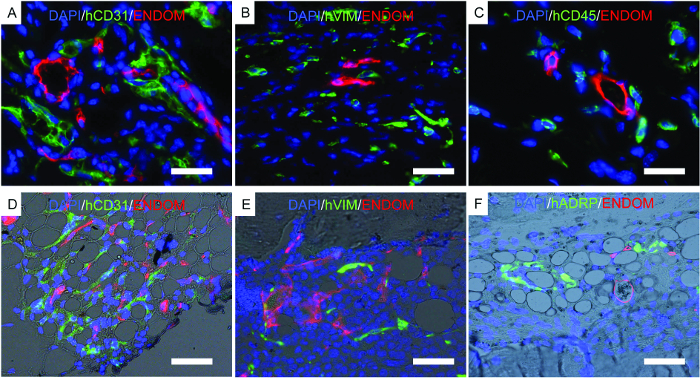

**Figure 7. Representative Images of Immunofluorescence Performed on Fixed Scaffolds.A-F)** Immunofluorescence studies performed to locate human cells implanted in scaffolds. **A-C) **Scaffolds implanted with hECs, hMSCs, and hHSCs. **D-F)** Bone-forming scaffolds implanted with hECs, hMSCs, and hHSCs. The color channels are as follows: **A-D) **human EC (hCD31) and mouse vascular structure (endomucin, ENDOM), **B-E)** hMSCs (hVimentin, hVIM) and mouse vascular cells (endomucin, ENDOM), **C)** human hematopoietic cells (hCD45) and mouse vasculature (endomucin, ENDOM), and **F)** human adipose differentiation-related protein (hADRP) and mouse vasculature (endomucin, ENDOM). Scale bars represent 10 µm (A and C), 20 µm (B and E), and 40 (D and F) µm. Please click here to view a larger version of this figure.

## Discussion

### Significance with Respect to Existing Methods:

In this protocol, we described a method to generate different humanized microenvironments in mice and to visualize their architecture via two-photon microscopy and histology. The representative data provided shows the feasibility of the approach, using different stromal cells to engineer humanized tissues. The protocol has specific applications to the study of human hematopoietic cells and bone marrow niche-derived cells in normal and pathological conditions. These applications include the study of clonal evolution, drug screening, and crosstalk between human HSCs and stromal components. In the emerging field of tissue engineering, several alternative approaches have been proposed. Approaches of note include the development of 3D humanized BM structures *in vitro*^55^,^56^,^57^,^58^,^59^,^60^,^61^,^62^,^63^ and the orthotopic graft of humanized BM scaffolds in mice^64^. Our approach has the advantage of combining both the complexity of the *in vivo* system with the easy anatomical accessibility of the humanized tissue graft.

### Modifications and Troubleshooting:

A source of variability in this protocol can be found in the selection of cells used to seed the scaffolds. In our work, we used BM-derived hMSCs. However, mesenchymal cells can be obtained from several tissues, which may show distinctive properties depending upon the origin. Therefore, the use of hMSCs derived from different organs can be considered. However, their ability to form bone tissue *in vivo* should be tested prior to use in this protocol.This protocol uses a commercially available human endothelial cell source (*i.e., *E4ORF1-transduced HUVEC). Recently, the use of organ-specific endothelial cells for different purposes has been reported^65^,^66^. Furthermore, the use of primary hECs derived from the BM could represent an interesting improvement to the protocol. Therefore, the use of different sources of endothelial cells may produce different *in vivo* outcomes.

We used NSG immunocompromised recipient mice to favor the implantation of humanized scaffolds and to avoid tissue rejection. We do not exclude the possibility of using this protocol to engineer ectopic bone marrow tissues in other mouse strains. Indeed, rhBMP-2 can induce bone formation in different mammalian models^47^,^48^,^49^,^50^,^52^. However, differences in cell viability and long-term transplantation are likely to be observed using different strains/models.

The timing of scaffold recovery can also be flexible, depending upon the final purpose of the experiment. In the presented protocol, we recover samples at 8 - 12 weeks after implantation to assess long-term hematopoietic engraftment. To study early steps of the human BM niche formation (*e.g.,* osteochondral tissue formation^47^ or vascular development), different time points can be chosen.

The live-imaging technique we described in this protocol is indicated for short-term imaging of explants. The use of an equilibrated chamber for maintaining physiological temperature, oxygen tension, and CO_2_ concentration should be considered in cases of long-term imaging, such as to study motility behaviors.

### Critical Steps within the Protocol:

Among the challenges related to the protocol, we would highlight the technical skills required for some steps. Mesenchymal and endothelial cells should be used at low cell passage numbers; otherwise, they will not be able to support human hematopoietic cell engraftment *in vivo* or to participate in *de novo* vasculature and bone formation *in vivo*. We recommend the use of hMSCs and hECs at passages 1 - 5. Scaffold preparation and cell-seeding steps require basic cell-culture skills and knowledge of the properties of the specific cells used in the procedure. The surgery protocol is quite straightforward but requires some practice. Maintenance of an aseptic environment to avoid contamination of the implanted scaffolds in immunodeficient mice is crucial to ensure the success of the experiment. Sample explant and live imaging require surgical practice (especially for the use of the microdrill) and knowledge of the microscope system. Finally, sample processing and histology require basic knowledge of the techniques to be used.

### Limitations of the Technique:

The approach we describe allows for the visualization of live human hematopoietic cells seeding a humanized bone marrow microenvironment, with human endothelial cells forming vascular structures and mesenchymal cells forming bone/bone marrow space. As the tissue is formed *in vivo*, the final engineered scaffold will still be chimeric (human and murine). This issue should be taken into account, as the chimeric tissue may not fully mimic human bone marrow complexity and environment.

The scaffolds implanted have a limited size (we tried a maximum of 6.6 x 7.5 x 7 mm), and therefore, they are able to host a limited number of cells for xenotransplantation. The absolute number of recovered cells will also be limited; thus, the number of implanted scaffolds should be calculated as a function of the number of cells required for the experiment.

The imaging application we described is particularly useful for observing large areas of live tissue at depths of 150-200 µm from the surface without disrupting the architecture and damaging the cells. Therefore, it does not allow for the visualization of the whole scaffold. If a complete scan of the tissue is required, standard immunofluorescence approaches would be more appropriate.

### Future Applications:

The future direction of this bioengineered model would be to increase the complexity of the human components in the tissue. The knowledge and characterization of the human BM niche has progressed in recent years^67^, and the described protocol could be an interesting platform to study the function of these new cellular components and soluble factors, as well as their role in supporting normal/malignant HSCs.

Furthermore, the imaging technique provides the potential for intravital imaging of the scaffolds in longitudinal studies, which would require technical improvements in imaging the scaffolds in live, anesthetized mice, with post-surgery recovery. This approach would require additional steps and is currently under investigation in the laboratory.

## Disclosures

The authors have nothing to disclose.
